# Incidence and determinants of neonatal morbidity after elective caesarean section at the national referral hospital in Kampala, Uganda

**DOI:** 10.1186/s13104-015-1617-7

**Published:** 2015-10-30

**Authors:** Annettee Nakimuli, Sarah Nakubulwa, Othman Kakaire, Michael O. Osinde, Scovia N. Mbalinda, Rose C. Nabirye, Nelson Kakande, Dan K. Kaye

**Affiliations:** Department of Obstetrics and Gynecology, School of Medicine, College of Health Sciences, Makerere University, P.O. Box 7072, Kampala, Uganda; Department of Obstetrics and Gynecology, Jinja Regional Hospital, Jinja, Uganda; Department of Nursing, School of Health Sciences, College of Health Sciences, Makerere University, P.O. Box 7072, Kampala, Uganda; Joint Clinical Research Centre, Clinical, Operations and Health Services Research Program, P. O. Box 10005, Kampala, Uganda

## Abstract

**Background:**

Elective caesarean sections (ECS) have been implicated in increased risk of adverse neonatal outcomes. The primary objective was to assess the incidence and determinants of neonatal morbidity after elective caesarean section deliveries. The secondary objective was to describe the maternal morbidity associated with elective caesarean section.

**Methods:**

This was a prospective cohort study of women admitted for ECS, as well as their newborns, conducted at Mulago hospital from March 1, 2013 to February 28, 2014. These were followed from the time of the operation until 6 weeks after hospitalization following the caesarean delivery. Data was collected using an interviewer-administered questionnaire and review of medical records for demographic characteristics, obstetric history, current pregnancy complications and pregnancy outcomes up to hospital discharge. Study outcomes were maternal and neonatal morbidity. The data was analyzed using Stata version 12.

**Results:**

There were 25,846 deliveries during the study period, of which 20,083 (77.7 %) were vaginal deliveries or assisted deliveries, and 5763 (22.3 %) were caesarean sections. Of the caesarean sections, 920 (15.9 %) were ECS. The commonest maternal morbidity was hemorrhage (17.2 %). A birth weight less than 2500 g (aRR 11.0 [95 % CI 8.1–17.2]) or more than 4000 g (aRR 12.2 [95 % CI 10.6–23.2]), delivery at gestation age less than or equal to 38 weeks (aRR 1.62 [95 % 1.20–2.10]), multigravidity (aRR 1.70 [95 % CI 1.20–2.90]) and using general anaesthesia (aRR 2.43 [95 % CI 1.20–5.90]) were associated with risk of neonatal morbidity. The commonest neonatal morbidity is respiratory distress especially if delivery occurs at a gestation age of 37 weeks or lower, if the birth weight is less than 2500 g or more than 4000 g, and if general anesthesia is used.

**Conclusion:**

Our study shows that at Mulago Hospital, ECS is associated with significant neonatal and maternal morbidity. We recommend that elective caesarean sections be performed after 39 weeks of gestation, and preferably avoid using general anaesthesia.

## Maternal and neonatal morbidity associated with elective caesarean section at the national referral hospital in Uganda

Cesarean deliveries are on the increase worldwide, partly due to an increase in primary caesarean deliveries and drop in the number of women attempting vaginal birth after a previous cesarean delivery (VBAC) [1]. Primary caesarean section refers to the caesarean section irrespective of the type of caesarean section or its indication, which includes caesarean section on maternal request or when there is no strong obstetric indication. This increases the number of caesarean sections from repeat caesarean section. This implies that women who have a primary cesarean section have a high chance of having a repeat cesarean section, which will increase the overall cesarean rate in the future. Although usually life-saving, and reducing maternal and neonatal morbidity and mortality from maternal or fetal complications, caesarean delivery is not without risks and may lead to or increase maternal and newborn morbidity [2]. A meta-analysis of nine studies [3] reported twice higher intrapartum and neonatal deaths among term, non-malformed infants who underwent a trial of labor compared to those who delivered by repeat elective cesarean (ECS). In contrast, a population-based study of neonatal and infant mortality stratified by mode of delivery among low-risk women found that neonatal mortality was more than doubled after a caesarean birth (as compared to vaginal delivery after trial of scar), even after excluding infants with congenital anomalies and birth asphyxia and adjusting for demographic and medical covariates [4]. Other studies reported similar findings of neonatal morbidity and mortality being common after ECS deliveries [5–7].

Since the risk of unexplained intrauterine fetal death increases as maternal gestation age rises (from 0.08 % at 38 weeks gestation to 0.34 % at 41 weeks gestation [8]), the neonatal risks associated with ECS must be considered in comparison to the competing risks of increased unexplained fetal death in ongoing pregnancy after 37 weeks [8–11] as well as maternal risks due to the operation. In both elective and emergency caesarean deliveries, intrapartum complications that can occur to the mother include infection, bleeding, visceral injury and anaesthetic complications [8–11], though infection is more likely after emergency deliveries. About 1–14 % of all patients undergoing caesarean delivery require blood transfusion [12]. Repeat caesarean section has a fourfold increased risk of visceral injury compared to primary caesarean section [13].

Caesarean section has been linked to adverse maternal or neonatal outcomes in several studies. Hillan [14] found that 77 % of women who delivered by ECS had at least three complications while 23 % had four or more, and febrile illness was the commonest complication in 48.2 % [14]. In another study [15], wound infection and puerperal febrile morbidity were the commonest complications, occurring in 1.5 % of patients. Silver et al. [16] reported a significantly increased risk of intensive care unit admission, hysterectomy, blood transfusion, ureteral injury, bowel injury and hospital stay as the number of caesarean delivery increases. Additionally, Hawkins et al. [17] found that 82 % of anesthesia-related maternal deaths occurred in women undergoing caesarean section, particularly from general anesthesia (52 % of 129 deaths). Another study of outcomes of caesarean deliveries in the United States found that surgery-related intraoperative complications (uterocervical and bladder lacerations, hemorrhage and gut injury) occurred in 12–15 % of caesarean births [18]. Postoperative morbidity associated with caesarean section includes wound infection, deep vein thrombosis, intrauterine infection, urinary tract infection, chest infection, febrile morbidity and postpartum hemorrhage [14–19].

While the neonatal morbidity could be assumed to be due to more high-risk pregnancies that are delivered operatively, particularly as emergency operations, there is scanty data on adverse outcomes after ECS in uncomplicated pregnancies in developing countries. A retrospective analysis of ECS at a Nigerian University hospital estimated the maternal complications associated with such delivery occurred in 53.7 %, of which need for blood transfusion and puerperal febrile morbidity contributed 11.6 and 11 % respectively [19]. ECS has been linked to adverse maternal and neonatal outcomes, including negatively impacting on bonding and early initiation of breastfeeding [20, 21]. It is critical to evaluate maternal and neonatal outcome associated with ECS, as well as their determinants. Data generated may be used in designing protocols and interventions to increase maternal and neonatal safety in relation to ECS. The primary objective therefore was to assess the incidence and determinants of neonatal morbidity after ECS. A secondary objective was to describe the maternal morbidity associated with ECS.

## Methods

### Study setting and design

This was a prospective cohort study of women admitted for ECS, as well as their newborns, who were followed for 6 weeks after hospitalization following the caesarean delivery from March 1, 2013 to February 28, 2014. The study was conducted at Mulago hospital, Uganda’s national referral hospital and the teaching hospital for Makerere University. The hospital has over 1500 beds, of which over 400 are maternity beds, and conducts over 30,000 deliveries per year, with over 40 obstetricians, over 50 trainee obstetricians, and more than 50 midwives attached to the maternity women. The neonatal intensive care unit (NICU) is manned by 3 neonatologists, 3 medical officers and over 30 nursing staff.

### Data collection

Women who consented to participate were recruited in the study on the day their names appeared on the theatre list for elective caesarean section. Using an interviewer-administered questionnaire, and through review of medical records, data was collected on demographic characteristics, obstetric history, current pregnancy complications and pregnancy outcomes up to hospital discharge. The study outcomes were adverse maternal and neonatal outcomes. The primary outcome (neonatal morbidity) was assessed by incidence of transient tachypnoea of the newborn, respiratory distress syndrome and persistent pulmonary hypertension, and management of serious respiratory morbidity [oxygen therapy for more than 12 h, nasal continuous positive airway pressure (CPAP) and need for mechanical ventilation], Apgar score at 5 min of less than 7, neonatal ward admission of more than 24 h, delayed initiation of breast feeding (more than 24 h), neonatal jaundice, neonatal sepsis and neonatal death. Maternal outcomes included operative blood loss, haemorrhage requiring blood transfusion, peripartum emergency hysterectomy, intraoperative visceral injury (extension of uterine incision, gut injury and bladder lacerations), anaesthetic complications (severe hypotension, insufficient duration of regional block and post-dural puncture headache), surgical wound infection or dehiscence, puerperal febrile morbidity (temperature ≥38 °C on two or more occasions on any 48 h period excluding the first 24 h postpartum), secondary postpartum haemorrhage, hospital stay of more than 5 days, admission to the high dependency unit and maternal death.

### Data analysis

We described maternal and neonatal complications and present them as frequencies and percentages for categorical variables. We analyzed risk factors for developing at least one neonatal complication after ECS. Categorical variables were compared with Chi square or Fisher’s exact test and continuous variables with a two-tailed student *t* test. Variables with a* p* < 0.2 were included in a Poisson regression model with robust variance to assess factors associated independently with risk of adverse neonatal outcomes. Results are expressed as risk ratios (RR) with 95 % confidence interval (CI). A *p* value of <0.05 was considered as significant.

### Ethical considerations

This research was part of a post-doctoral research project of the first author (DKK) on *Evaluation and surveillance of the impact of maternal and neonatal near*-*miss morbidity on the health of mothers and infants*. Ethical approval to conduct the study was obtained from the Ethics and research committees of Mulago hospital, the School of Medicine, Makerere University College of Health Sciences and Uganda National Council for Science and Technology. Permission to conduct the study was obtained from the department of Obstetrics and Gynaecology, Makerere University. All participants gave written informed consent to be interviewed.

## Results

There were 25,846 deliveries during the study period, of which 20,083 (77.7 %) were vaginal deliveries, 5763 (22.3 %) were caesarean section of which 920 (16.0 %) were ECS for singleton pregnancies. In Table 1, the ages of the women are shown. The mean age of the women who had ECS was 31.6 ± 4.8 years, with the majority being 26–35 years (55.7 %). Most had attained at least secondary level of education.

**Table 1:** Socio-demographic and obstetric characteristics of the study population

Characteristic	N = 920	Percentage
Age (years)		
16–25	380	41.3
26–35	512	55.7
More than 35	28	3.0
Education level		
No formal education	13	1.3
Primary education	369	40.2
Secondary education	408	44.3
Tertiary education	130	14.1
Marital status		
Married	624	67.8
Single	248	26.9
Cohabiting	38	4.1
Divorced/widowed	10	1.1
Occupation		
Formally employed	380	41.5
Unemployed or fulltime housewife	308	33.3
Business woman or employed in informal sector	210	22.8
Others	22	2.4
Parity		
1	334	36.3
2–4	462	50.2
5 and above	124	13.2
Gestation age at the time of caesarean delivery (weeks)		
Less than 37	30	3.2
37–38	600	65.2
>38	290	31.6

In Table 2, the commonest indication for ECS was one previous caesarean Section (45.7 %), while for 13.9 %, the indication was two or more previous caesarean scars. One in every 5 women (20.0 %) suffered at least one maternal complication, mainly hemorrhage with blood loss of more than 1000 ml (17.2 %); and more than 11 % were hospitalized for longer than 5 days.

**Table 2:** The main indication and complications of elective caesarean section

Indication	N = 920^a^	Percentage
One previous c/s scar	420	45.7
Two or more previous c/s scars	128	13.9
Poor obstetric history	96	10.4
Hypertensive disorders	52	5.7
Big baby	28	3.0
Contracted pelvis	16	1.7
Breech and other malpresentation	32	3.5
Post term	24	2.6
Cord around the neck	20	2.2
Uterine pathology	8	1.7
Elderly primigravidae	7	1.5
Medical diseases	7	1.5
Placenta previa	4	0.9
Infertility with poor obstetric history	6	1.3
Others	25	3.0
Maternal complications		
Blood loss ≥ 1000 ml	158	17.2
Extension of uterine incision	60	6.5
Pyrexia (febrile morbidity)	44	4.7
Blood transfusion necessitated by blood loss	32	3.4
Anaesthetic complications	16	1.7
Wound sepsis or dehiscence	28	3.0
Urinary bladder injury requiring catheterization longer than 24 h	12	1.3
Peripartum hysterectomy	4	0.9
Hospitalization for longer than 7 days	104	11.1

^a^Other causes include congenital abnormalities, unclear indications and maternal request as reasons for the caesarean section; *c/s* caesarean section

In Table 3, most neonates (60 %) were admitted to neonatal ward for observation. The mean birth weight was 3130 ± 560 g. However, 116 (12.6 %) babies developed at least one complication that necessitated admission to the NICU, and 26 (2.8 %) were admitted for more than 48 h in the NICU.

**Table 3:** Neonatal outcomes for women who had elective caesarean section

Characteristic	Frequency	Percentages
Sex of the baby		
Male	450	49.0
Female	470	51.0
Birth weight of the baby in kg		
<2.5	94	10.2
2.5–3.99	808	87.8
≥4	18	2.0
Initiation of breastfeeding		
Within 24 h	260	28.3
≥24 h	660	71.7
Admission to the NICU		
Yes	670	72.8
No	250	27.2
Reason of admission (670)		
Grunting respiration	160	72.8
Low Apgar score (less than 7 at 5 min)	78	27.2
Prematurity	20	23.8
Others	10	1.0
Observation^a^	402	60.0
Neonatal complications (n = 116)^b^		
Neonatal sepsis	38	32.7
Adverse respiratory outcomes^c^	68	58.6
Admission for more than 48 h in NICU	26	22.4
Early neonatal death	4	3.4
Low apgar score (less than 4) at 5 min	30	25.9
Neonatal jaundice	16	13.8
X-ray suggestive of alveolar disease^d^	26	22.4

^a^Observation was the indication for admission to NICU for macrosomic babies and babies where the operation was due to a maternal or fetal indication

^b^Some babies had more than one complication

^c^Adverse respiratory outcomes include pulmonary hypertension, transient tachypnoea, apneic attacks or respiratory distress syndrome that developed features of progressive respiratory distress occurring shortly after birth, with grunting respiration, retractions during inspiration, cyanosis, and reduced or absent breathing sounds

^d^X-rays were done only on babies that did not improve in 48 h

In Table 4, a birth weight of less than 2500 g or more than 4000 g, delivery at a gestation age of 38 weeks or earlier, multiparity and use of general anaesthesia were independently associated with neonatal adverse outcomes.

**Table 4:** Factors associated with adverse neonatal outcome

Characteristic	Total	Adverse outcome		Relative risk	Adjusted relative risk
		Present N (%) n = 116	Not present N (%) n = 804		
Birth weight of babies in kg					
<2.5	94	58 (50.0)	36 (4.5)	10.8 (7.8–15.0)	11.0 (8.1–17.2)
2.5–3.99	808	46 (39.7)	762 (94.7)	Ref	Ref
≥4	18	12 (10.3)	6 (0.7)	11.7 (7.6–18.3)	23.2 (11.6–47.2)
Sex of baby					
Male	450	57 (49.1)	393 (48.9)	Ref	Ref
Female	470	59 (50.1)	411 (51.1)	1.0 (0.7–1.4)	1.40 (0.80–2.10)
Gestational age at delivery in weeks					
≤38	690	77 (66.3)	613 (76.3)	1.5 (1.1–2.2)	1.60 (1.20–2.10)
>38	230	39 (33.7)	191 (23.6)	Ref	Ref
Parity					
Primigravida	320	50 (43.1)	270 (33.6)	Ref	Ref
Multigravida	600	66 (56.8)	534 (66.4)	1.4 (1.0–2.0)	1.70 (1.20–2.90)
Indication for c/s					
Maternal indication	150	22 (18.9)	138 (17.2)	0.87 (0.58–1.37)	0.93 (0.62–1.36)
Fetal indication	222	37 (31.8)	185 (23.0)	Ref	Ref
Previous caesarean section	548	57 (57.8)	481 (61.1)	0.98 (0.85–1.12)	0.76 (0.54–1.66)
Anaesthesia used					
Spinal	433	23 (19.8)	410 (50.9)	Ref	Ref
General	487	93 (80.1)	394 (49.0)	3.6 (2.3–5.6)	2.4 (1.2–5.7)

*Ref* reference group, *c/s* caesarean section

## Discussion

The study shows that the commonest indication for ECS was repeat caesarean section. The commonest maternal morbidity was hemorrhage, more than every tenth woman required hospitalization longer than 5 days, and respiratory complications were the commonest neonatal morbidity.

Previous studies showed that ECS was associated with an increased risk of respiratory morbidity in neonates [22], especially from preterms babies, where the main cause is surfactant deficiency. This morbidity results from relative absence of hormonal and physiological changes associated with labour which are necessary lung function in neonates, and which do partially occur in newborns delivered as preterms [23–25]. The changes that occur in the fetal lungs in preparation for delivery include an increase in the small pulmonary blood vessels (up to 40 times) in the third trimester, changes in the epithelial sodium channels with increased ability to clear foetal lung fluid at term, and decrease of chloride channels which lead to decrease of fluid secretion [23–25]. The gestational age at the time of ECS may thus be a critical determinant of the risk for this respiratory morbidity in neonates [26], which occurs usually after 48 h of birth [23–25].

In addition, the maturation and changes that occur during labor play an important role in reducing respiratory distress and transient tachypnoea of the newborn [23–26]. Respiratory distress syndrome (RDS) may occur in term babies, particularly after ECS [27]. It differs from preterm RDS regarding earlier onset [27], more frequent occurrence in male babies, more frequent association with birth asphyxia, pulmonary hemorrhage, perinatal infections (such as septicaemia), and multiple organ-system failure [27]. Term neonatal RDS is also associated with higher need for oxygen and mechanical ventilation, more prolonged hospitalization and higher mortality than other causes of respiratory morbidity in term babies. It is possible that many babies in our study developed term RDS, as a large number had delivery between 37 and 38 weeks of gestation, had low 5-min Apgar scores, required mechanical ventilation, required CPAP, required NICU admission for more than 48 h. Results of the present study confirm the observation that the newborn transition from a fluid filled lung to a lung filled with air in a very short period of time, as happens in ECS, constitutes a big challenge to some newborns, resulting into respiratory morbidity where there is failure to clear fetal lung fluid.

Factors associated with RDS in term babies include ECS, where serious respiratory morbidity is associated with decreasing gestational age, due to relative surfactant deficiency [28]. Other causes include severe birth asphyxia, neonatal septicemia, meconium aspiration syndrome and pulmonary hemorrhage [28]. These are unlikely in neonates delivered by ECS.

The present study are in agreement with in the 2005 WHO global survey on maternal and perinatal health in Latin America, which found that maternal morbidity was common after ECS [29], and that caesarean delivery was associated with an increase in fetal morbidity and admissions to the NICU for 7 days or longer even after adjustment for preterm delivery [29]. Tita et al. [7] found more neonatal deaths, respiratory complications, hypoglycemia, newborn sepsis, and admissions to the NICU for ECS done at less than 39 weeks of gestation compared to ECS after 39 weeks. The present study reinforces the observation that neonatal morbidity may be reduced if ECS are performed under spinal anesthesia, and after 39 weeks of gestation.

The present study also shows that maternal morbidity is common after ECS, as one in every five women had complications, most commonly hemorrhage. The maternal risk of hemorrhage in ECS stems from suboptimal development of the lower uterine segment or from adhesions, both of which increase intraoperative blood loss [25, 26, 30–32]. The risk of difficult surgery, visceral injury and prolonged hospitalization is common in such patients. However, postponing repeat cesarean delivery until 39 weeks increases the chance that the operation is performed unscheduled as an emergency or after onset of labor [25, 26, 30], as up to 25 % of pregnant women go into labor between 38 and 39 weeks [33]. Moreover, repeat cesarean sections performed as emergencies (after the onset of labor) carry higher risks of complications such as uterine rupture, infection and maternal mortality than elective procedures.

The findings of the study have implications for counseling women on decision-making for mode of delivery. Women with a previous caesarean section for a non-permanent indication have to choose between a vaginal birth (VBAC) and ECS. Each of these has inherent risks. Short-term maternal risks related to VBAC such as uterine rupture are potentially catastrophic. In addition, there is in addition increased risk of perinatal mortality and birth asphyxia in newborns delivered by emergency caesarean section after a failed trial of labor. These risks compete with the risks associated with ECS, particularly maternal hemorrhage and neonatal respiratory morbidity [34–37]. Therefore, the risks and benefits associated with a specific mode of delivery (VBAC or ECS for eligible women) at a given gestational age should be compared with the potential consequences of pregnancy continuation beyond that time point (which may include sudden unexplained fetal demise and onset of spontaneous labor). This is particularly relevant when spontaneous labor occurs in women with placenta previa, placenta accreta, prior classical cesarean delivery or prior myomectomy [32, 33, 38]. The findings call for provision of patient-centred care where mothers are involved in the decision-making whether or not to have a trial of scar or an elective caesarean delivery.

## Conclusion

Our study shows that at Mulago Hospital, elective caesarean delivery is associated with significant neonatal and maternal morbidity. The commonest neonatal morbidity is respiratory distress especially if delivery occurs at 38 weeks of gestation or earlier, if the birth weight is less than 2500 g or more than 4000 g, and if general anesthesia is used.

## References

[1] Bragg F, Cromwell DA, Edozien LC, Gurol-Urganci I, Mahmood TA, Templeton A, van der Meulen JH (2010). Variation in rates of caesarean section among English NHS trusts after accounting for maternal and clinical risk. BMJ.

[2] Villar J, Carroli G, Zavaleta N, Donner A, Wojdyla D, Faundes A (2007). Maternal and neonatal individual risks and benefits associated with caesarean delivery; multicentre prospective study. BMJ.

[3] Mozurkewich EL, Hutton EK (2000). Elective repeat cesarean delivery versus trial of labor: a meta-analysis of the literature from 1989 to 1999. Am J Obstet Gynecol.

[4] Richardson BS, Czikk MJ, daSilva O, Natale R (2005). The impact of labor at term on measures of neonatal outcome. Am J Obstet Gynecol.

[5] De Luca R, Boulvain M, Irion O, Berner M, Pfister RE (2009). Incidence of early neonatal mortality and morbidity after late-preterm and term cesarean delivery. Pediatrics.

[6] Hook B, Kiwi R, Amini SB, Fanaroff A, Hack M (1997). Neonatal morbidity after elective repeat cesarean section and trial of labor. Pediatrics.

[7] Tita AT, Landon MB, Spong CY, Lai Y, Leveno KJ, Varner MW (2009). Timing of elective repeat cesarean delivery at term and neonatal outcomes. N Engl J Med.

[8] Smith GC, Pell JP, Cameron AD, Dobbie R (2002). Risk of perinatal death associated with labor after previous cesarean delivery in uncomplicated term pregnancies. JAMA.

[9] Froen JF, Arnestad M, Frey K, Vege A, Saugstad OD, Stray-Pedersen B (2001). Risk factors for sudden intrauterine unexplained death: epidemiologic characteristics of singleton cases in Oslo, Norway, 1986–1995. Am J Obstet Gynecol.

[10] Yudkin PL, Wood L, Redman CW (1987). Risk of unexplained stillbirth at different gestational ages. Lancet.

[11] Smith GC (2001). Life-table analysis of the risk of perinatal death at term and post term in singleton pregnancies. Am J Obstet Gynecol.

[12] Naef RW, Washburne JF, Martin RW, Magann EF, Scanlon PH, Morrison JC (1995). Hemorrhage associated with cesarean delivery: when is transfusion needed?. J Perinatol.

[13] Declercq E, Barger M, Cabral HJ, Evans SR, Kotelchuck M, Simon C (2007). Maternal outcomes associated with planned primary cesarean births compared with planned vaginal births. Obstet Gynecol.

[14] Hillan EM (1995). Postoperative morbidity following Caesarean delivery. J Adv Nurs.

[15] van den Berg A, van Elburg RM, van Geijn HP, Fetter WP (2001). Neonatal respiratory morbidity following elective caesarean section in term infants. A 5-year retrospective study and a review of the literature. Eur J Obstet Gynecol Reprod Biol.

[16] Silver RM, Landon MB, Rouse DJ, Leveno KJ, Spong CY, Thom EA (2006). Maternal morbidity associated with multiple repeat cesarean deliveries. Obstet Gynecol.

[17] Hawkins JL, Koonin LM, Palmer SK, Gibbs CP (1997). Anaesthesia related deaths during obstetric delivery in the United States 1979-90. Anaeshtesiology.

[18] van Ham MA, van Dongen PW, Mulder J (1997). Maternal consequences of cesarean section: a retrospective study of intraoperative and postoperative maternal complications of cesarean section during a 10 year period. Eur J Obstet Gynecol Reprod Biol.

[19] Oladapo OT, Lamina MA, Sule-Odu AO (2007). Maternal morbidity and mortality associated with elective caesarean delivery at a university hospital in Nigeria. Aust N Z J Obstet Gynaecol.

[20] Leung GM, Lam TH, Ho LM (2002). Breast-feeding and its relation to smoking and mode of delivery. Obstet Gynecol.

[21] Rowe-Murray HJ, Fisher JR (2002). Baby friendly hospital practices: cesarean section is a persistent barrier to early initiation of breastfeeding. Birth.

[22] Hansen AK, Wisborg K, Uldbjerg N, Henriksen TB (2007). Elective caesarean section and respiratory morbidity in the term and near-term neonate. Acta Obstet Gynecol Scand.

[23] Madar J, Richmond S, Hey E (1999). Surfactant-deficient respiratory distress after elective delivery at “term”. Acta Paediatr.

[24] Jain L, Eaton DC (2006). Physiology of fetal lung fluid clearance and the effect of labor. Semin Perinatol.

[25] Morrison JJ, Rennie JM, Milton PJ (1995). Neonatal respiratory morbidity and mode of delivery at term: influence of timing of elective caesarean section. Br J Obstet Gynaecol.

[26] Chiossi G, Lai Y, Landon MB, Spong CY, Rouse DJ, Varner MW (2013). Timing of delivery and adverse outcomes in term singleton repeat cesarean deliveries. Obstet Gynecol.

[27] Liu J (2014). Respiratory distress syndrome in full-term neonates. J Neonatal Bio.

[28] Liu J, Shi Y, Dong JY, Zheng T, Li JY, Lu LL (2010). Clinical characteristics, diagnosis and management of respiratory distress syndrome in full-term neonates. Chin Med J (Engl)..

[29] Villar J, Valladares E, Wojdyla D, Zavaleta N, Carroli G, Velazco A (2006). Caesarean delivery rates and pregnancy outcomes: the 2005 WHO global survey on maternal and perinatal health in Latin America. Lancet.

[30] Salim R, Shalev E (2010). Health implications resulting from the timing of elective cesarean delivery. Reprod Biol Endocrinol..

[31] Nouaili BHE, Bouziri A (2010). Ben Miled A, Chaouachi S, Sfar R, Ben Jaballah N. Neonatal respiratory morbidity after elective cesarean section at term. Tunis Med..

[32] Hansen AK, Wisborg K, Uldbjerg N, Henriksen TB (2008). Risk of respiratory morbidity in term infants delivered by elective cesarean section: a cohort study. BMJ.

[33] Rossi AC, D’Addario V (2008). Maternal morbidity following a trial of labor after cesarean section Versus elective repeat cesarean delivery: a systematic review with metaanalysis. Am J Obstet Gynecol..

[34] Patel RM, Jain L (2010). Delivery after previous cesarean: short-term perinatal outcomes. Semin Perinatol..

[35] Hansen AK, Wisborg K, Uldbjerg N, Henriksen TB (2007). Elective caesarean section and respiratory morbidity in the term and near-term neonate. Acta Obstet Gynecol Scand..

[36] Zanardo V, Simbi AK, Franzoi M, Soldà G, Salvadori A, Trevisanuto D (2004). Neonatal respiratory morbidity risk and mode of delivery at term: influence of timing of elective caesarean delivery. Acta Paediatr..

[37] Sigalas J, Galazios G, Tsikrikoni I, Scordala M, Vogiatjaki T, Spanopoulou PI (2006). The influence of the mode of anaesthesia in the incidence of neonatal morbidity after an elective caesarean section. Clin Exp Obstet Gynecol..

[38] Allen VM, O’Connell CM, Baskett TF (2006). Maternal morbidity associated with cesarean delivery without labor compared with induction of labor at term. Obstet Gynecol..

